# Effect of aspirin on healthspan in community-dwelling older adults: the ASPirin in reducing events in the elderly study

**DOI:** 10.1093/ageing/afag218

**Published:** 2026-07-24

**Authors:** Haoxin Tina Zheng, Aung Zaw Zaw Phyo, Zimu Wu, Suzanne G Orchard, Alice Owen, Sara E Espinoza, Kerry Sheets, Robyn Woods, Joanne Ryan

**Affiliations:** School of Public Health and Preventive Medicine, Monash University School of Public Health and Preventive Medicine, 503 St Kilda Rd. Melbourne VIC 3004 Australia; School of Public Health and Preventive Medicine, Monash University School of Public Health and Preventive Medicine, 503 St Kilda Rd. Melbourne VIC 3004 Australia; School of Public Health and Preventive Medicine, Monash University School of Public Health and Preventive Medicine, 503 St Kilda Rd. Melbourne VIC 3004 Australia; School of Public Health and Preventive Medicine, Monash University School of Public Health and Preventive Medicine, 503 St Kilda Rd. Melbourne VIC 3004 Australia; School of Public Health and Preventive Medicine, Monash University School of Public Health and Preventive Medicine, 503 St Kilda Rd. Melbourne VIC 3004 Australia; Division of Geriatrics, Gerontology and Palliative Medicine, UT Health San Antonio, San Antonio, Texas, USA; Division of Geriatric and Palliative Medicine, Department of Medicine, Hennepin Healthcare, Minneapolis, Minnesota, USA; Berman Center for Outcomes and Clinical Research, Hennepin Healthcare Research Institute, Minneapolis, Minnesota, USA; School of Public Health and Preventive Medicine, Monash University School of Public Health and Preventive Medicine, 503 St Kilda Rd. Melbourne VIC 3004 Australia; School of Public Health and Preventive Medicine, Monash University School of Public Health and Preventive Medicine, 503 St Kilda Rd. Melbourne VIC 3004 Australia

**Keywords:** healthspan, aspirin, randomized controlled trial, older people

## Abstract

**Background:**

Inflammation is a shared pathogenic pathway underlying multiple chronic conditions and contributes to a loss of healthspan. This study aimed to evaluate the effect of low-dose aspirin on the loss of healthspan in older individuals.

**Methods:**

ASPirin in Reducing Events in the Elderly (ASPREE) was a double-blind, placebo-controlled, randomised trial assessing the effect of daily low-dose aspirin for primary prevention. Community-dwelling participants aged ≥70 years [or ≥ 65 years for United States (US) minority ethnic groups] were recruited from Australia and the US. Eligible participants were initially free of major diseases with a life expectancy of more than five years and were randomly assigned to receive 100 mg of aspirin daily or a placebo. Loss of healthspan was defined as the first occurrence of cardiovascular disease, diabetes, dementia, cancer, or death.

**Results:**

A total of 19 114 participants were followed for a median of 4.7 years, 5835 participants (30.5%) experienced a loss of healthspan. Participants who were older, male, smokers, or had hypertension, or frailty were more likely to experience healthspan loss during the trial. The rate of healthspan loss was 82.1 and 82.7 events per 1000 person-years in the aspirin and placebo groups, respectively. There was no evidence of a substantial difference in the risk of healthspan loss between the aspirin and placebo groups (hazard ratio, 0.98; 95% CI: 0.92–1.05).

**Conclusion:**

Low-dose aspirin had no effect on the rate of loss of healthspan among older community-dwelling adults. Future studies should explore lifestyle and behavioural interventions to extend healthspan.

## Key Points

- Daily intake of aspirin does not have any effect on prolonging healthspan.
- Older age, male sex, smoking, hypertension, and frailty were associated with an increased risk of healthspan loss.
- Future research could consider interventions that target integrated pathways of ageing to extend healthspan.

## Background

The global population of older adults has grown dramatically in recent decades [1, 2], driven by an increase in human lifespan [3, 4]. However, this increase has not been matched by a similar expansion of healthspan, the period of life lived in good health [3, 5, 6]. As a result, many older individuals are living longer with chronic diseases and disabilities [3], impacting physical independence, mental wellbeing, and overall quality of life [7, 8]. Identifying effective interventions to extend healthspan is imperative to support healthy ageing [6, 9].

Inflammatory pathways have been implicated in many chronic diseases associated with ageing including cardiovascular disease (CVD), cancer, diabetes and dementia [10–13]. For example, CVD develops from atherosclerosis, a condition in which inflammation plays a central role in formation and progression [14]. Chronic inflammation is also linked to tumour development, cancer initiation and progression via several pathways [10, 15]. Similarly, inflammation mediates the relationship between impaired insulin production and the onset of diabetes via an immune-driven pathway [11, 12]. Neuroinflammation contributes to cognitive decline through cell damage and possible genetic mutations, ultimately leading to neurodegeneration and dementia [13]. Given that inflammation underlies the development of many chronic diseases, anti-inflammatory drugs have potential as preventive therapies to delay their onset and extend healthspan [10, 11, 13, 16–20].

Aspirin is commonly used for the secondary prevention of CVDs [21, 22]. Aspirin may also exert cancer-preventive effects mainly through the inhibition of cyclooxygenase (COX) enzymes [23, 24]. Its anti-inflammatory and antiplatelet effects are mediated primarily through the acetylation of COX-2 and the inhibition of COX-1 [25, 26]. Thus, aspirin may provide protective effects on healthspan via modulation of inflammatory processes and/or through cardiovascular benefits.

Some previous observational studies have reported associations between the use of low-dose aspirin and a reduced disease risk [2, 17, 18, 27, 28]. However, large population-based randomised controlled trials have produced heterogeneous findings regarding low-dose aspirin’s effect on CVD [29–31], cancer [32, 33], dementia [34, 35], diabetes [2, 36, 37], and mortality [38–40]. For example, aspirin may reduce the risk of diabetes [2] but increase cancer-related deaths among older individuals [32]. Aspirin’s potential benefits may also be offset by its known bleeding risk [41]. The net effect of low-dose aspirin on a composite measure capturing healthspan is unknown. The aim of this study was therefore to evaluate the impact of daily low-dose aspirin intake versus placebo on healthspan, defined as life years lived without major diseases [42].

## Methods

### Study design and participants

Aspirin in Reducing Events in the Elderly (ASPREE) was a large, randomised, double-blind, placebo-controlled trial designed to evaluate the effect of daily low-dose aspirin versus placebo on prolonging independent life [38]. This trial recruited 19 114 relatively healthy, community-dwelling older individuals from Australia and the United States (US) between March 2010 and December 2014. At recruitment, all participants were free of dementia, CVD, physical disability, or conditions that were likely to cause death within 5 years. Eligible individuals, aged at least 70 years (or 65 years for US minorities), were randomly assigned to 100 mg enteric-coated aspirin daily or placebo.

During the trial period, participants attended in-person annual study visits and were contacted by telephone regularly to monitor adherence, record any adverse events, and identify any potential endpoints. The median follow-up time was 4.7 years. The intervention ceased in June 2017 following a futility analysis for the composite primary endpoint of disability-free survival, defined as survival free from dementia, or persistent physical disability. A comprehensive trial protocol, including details of study design, outcome definitions, and data collection procedures, has been published previously [43, 44].

### Trial registration and participant consent

The ASPREE trial received ethical approval from institutional review boards and ethics committees in both Australia and the US, as well as oversight from the U.S. National Institutes of Health. All participants provided written informed consent prior to enrollment. The study was conducted in accordance with the principles of the Declaration of Helsinki and Good Clinical Practise guidelines. ASPREE was registered with the International Standard Randomized Controlled Trial Number Register (ISRCTN83772183) and ClinicalTrials.gov (NCT01038583).

### Outcome measure

While there is no single definition of healthspan, the period of life spent without major chronic disease is one of the most frequently used [45–48]. Zenin *et al*. identified major chronic diseases that were most significantly associated with increasing age and mortality using data from the UK Biobank cohort. These conditions were: diabetes, congestive heart failure, myocardial infarction (MI), stroke, dementia, cancer, chronic obstructive pulmonary disease (COPD) and death [42]. Numerous subsequent studies have also applied this definition [46–49]. In line with this research [42], we also defined loss of healthspan as the first occurrence of diabetes, CVD (including MI, stroke and heart failure), dementia, cancer, and death. These outcomes were chosen because they are common major age-related conditions that substantially linked to morbidity, mortality, reduced quality of life and increased demand on healthcare systems [42]. The use of first occurrence captures the early-stage clinical transition from healthy ageing to loss of healthspan. COPD was not included due to a lack of data from the ASPREE trial [43]. Details on adjudication of each of the conditions included in loss of healthspan are described in the Supplementary Data section, Appendix 1.

### Statistical analysis

The effect of daily low-dose aspirin on time to loss of healthspan was examined using an intention-to-treat approach. Time to loss of healthspan was defined as the date of first occurrence of diabetes, dementia, CVD, cancer or death. All participants were included in the analysis. Participants with prevalent diabetes at baseline contributed zero person-years to the time-to-loss-of-healthspan analysis.

A Cox proportional hazards regression model was employed to compare time to loss of healthspan between the aspirin and placebo groups. Year 7 follow-up was excluded due to the small number of participants reaching this time point (*n* < 100). Proportional hazards assumptions were checked using visual inspection of Schoenfeld residuals. Potential heterogeneity in the effect of aspirin on healthspan was assessed by stratified subgroup analysis with interaction term included in the Cox model. Prespecified subgroups included age, sex, ethnicity, body mass index (BMI), smoking status, prevalent diabetes, hypertension status, dyslipidemia, cancer history, and previous aspirin use [44]. Cumulative incidence curves were generated using competing risks regression to visualise the risk of healthspan loss over time by treatment group. Three sensitivity analyses were performed: (i) excluding participants with prevalent diabetes, (ii) adding persistent physical disability to the endpoint (defined as inability/severe difficulty in performing at least one of the six basic activities of daily living, which persisted for at least 6 months, or loss of physical function independence [50] [51]), (iii) removing diabetes from the outcome definition. All analyses were conducted using Stata 17.0 (StataCorp, College Station, Texas).

## Results

**Figure 1:**
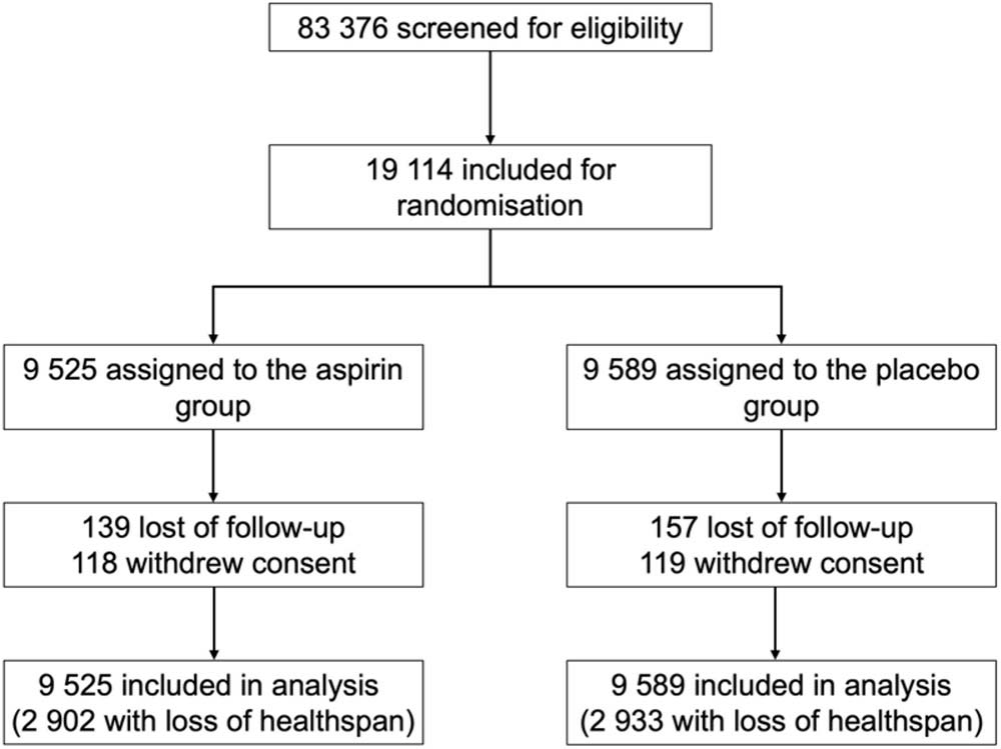
Study flow diagram.

There were 19 114 participants randomised (9525 to aspirin, 9589 to placebo group) and included in the analysis (Figure 1). Overall, demographic and lifestyle characteristics were well-balanced between the two randomisation groups (Table 1).

**Table 1:** Participant characteristics at baseline by randomisation groups.

		Placebo	Aspirin
Age, median [IQR]		73.95 [71.6, 77.6]	74.04 [71.6, 77.8]
Sex, n (%)	Female	5409 (56.4)	5373 (56.4)
Ethnicity, n (%)	White Australians	8193 (85.4)	8169 (85.8)
	White Americans	549 (5.7)	539 (5.7)
	African American	450 (4.7)	451 (4.7)
	Hispanic/Latino	248 (2.6)	240 (2.5)
	Other	149 (1.6)	126 (1.3)
BMI, n (%)	Underweight	176 (1.8)	185 (2.0)
	Normal weight	2287 (24.0)	2316 (24.4)
	Overweight	4263 (44.7)	4189 (44.2)
	Obese	2817 (29.5)	2792 (29.4)
Education, n (%)	<12 years	4329 (45.1)	4307 (45.2)
	12–15 years	2772 (28.9)	2802 (29.4)
	16+ years	2488 (25.9)	2415 (25.4)
Smoking status, n (%)	Current	383 (4.0)	352 (3.7)
	Former	3890 (40.6)	3909 (41.0)
	Never	5316 (55.4)	5264 (55.3)
Alcohol consumption, n (%)	Current	7333 (76.5)	7309 (76.7)
	Former	570 (5.9)	566 (5.9)
	Never	1686 (17.6)	1650 (17.3)
Living alone, n (%)		3154 (32.9)	3097 (32.5)
Prevalent diabetes, n (%)		1021 (10.6)	1024 (10.8)
Hypertension, n (%)		7131 (74.4)	7047 (74.0)
Dyslipidemia, n (%)		6306 (65.8)	6161 (64.7)
Frailty, n (%)	Not frail	5639 (58.8)	5606 (58.9)
	Pre-frail	3742 (39.0)	3705 (38.9)
	Frail	208 (2.2)	214 (2.2)

IQR: interquartile range; BMI: Body mass index; BMI range: Underweight: <20 kg/m^2^, Normal weight: 20–24.9 kg/m^2^, Overweight: 25–29.9 kg/m^2^, Obese: >30 kg/m^2^. Definition of frailty status was classified as not-frail: no criteria met, pre-frail: 1 or 2 criteria met, and frail: 3 or more criteria of the adapted Fried frailty criteria including body weight, strength, exhaustion, walking speed and physical activity [44]. In the race/ethnicity category, ‘Other’ includes participants who identified as Aboriginal or Torres Strait Islander, Native American, Native Hawaiian or Pacific Islander, mixed race, or those who were non-Hispanic and did not specify their race or ethnicity. Hypertension was defined as current use of antihypertensive medication or a blood pressure measurement exceeding 140/90 mmHg at study entry. Dyslipidemia was defined as taking lipid-lowering medication or having a serum cholesterol level ≥ 212 mg/dL (≥5 mmol/L in Australia) or ≥ 240 mg/dL (≥6.2 mmol/L in the US), or a low-density lipoprotein cholesterol level > 160 mg/dL (>4.1 mmol/L).

There were 5835 (30.5%) participants who had loss of healthspan during a total of 70 813 person-years, with an average of 4.7 follow-up years of the trial (see Table 2 and Supplementary Data Appendix 2).

**Table 2:** Participant characteristics at baseline by healthspan outcomes.

		Without loss of healthspan	Loss of healthspan
Number of participants, n (%)		13,279 (69.5)	5835 (30.5)
Age, median [IQR]		73.66 [71.5, 77.1]	74.79 [71.9, 79.0]
Females, n (%)		7963 (60.0)	2819 (48.3)
Ethnicity, n (%)	White Australian	11,500 (86.6)	4862 (83.3)
	White American	773 (5.8)	315 (5.4)
	African American	528 (4.0)	373 (6.4)
	Hispanic/Latino	305 (2.3)	183 (3.1)
	Other	173 (1.3)	102 (1.8)
BMI, n (%)	Underweight	258 (2.0)	103 (1.8)
	Normal weight	3403 (25.8)	1200 (20.7)
	Overweight	6049 (45.8)	2403 (41.4)
	Obese	3505 (26.5)	2104 (36.2)
Years of Education, n (%)	<12	5870 (44.2)	2766 (47.4)
	12–15	3851 (29.0)	1723 (29.5)
	16+	3557 (26.8)	1346 (23.1)
Smoking status, n (%)	Current	432 (3.3)	303 (5.2)
	Former	5228 (39.4)	2571 (44.1)
	Never	7619 (57.4)	2961 (50.8)
Alcohol consumption, n (%)	Current	10,373 (78.1)	4269 (73.2)
	Former	678 (5.1)	458 (7.9)
	Never	2228 (16.8)	1108 (19.0)
Living alone		4229 (31.8)	2022 (34.7)
3MS score, Mean (SD)		93.78 (4.4)	92.59 (4.9)
Previous aspirin use		1288 (9.7)	806 (13.8)
History of cancer		2454 (18.5)	1230 (21.1)
Hypertension		9500 (71.5)	4678 (80.2)
Dyslipidemia		8630 (65.0)	3837 (65.8)
Frailty status	Not frail	8310 (62.6)	2935 (50.3)
	Pre-frail	4739 (35.7)	2708 (46.4)
	Frail	230 (1.7)	192 (3.3)

IQR: interquartile range; SD: standard derviation; BMI: Body mass index; 3MS: Modified Mini-Mental State examination. BMI range: Underweight: <20 kg/m^2^, Normal weight: 20–24.9 kg/m^2^, Overweight: 25–29.9 kg/m^2^, Obese: >30 kg/m^2^. Definition of frailty status was classified as not-frail: no criteria met, pre-frail: 1 or 2 criteria met, and frail: 3 or more criteria of the adapted Fried frailty criteria including body weight, strength, exhaustion, walking speed and physical activity [44]. In the race/ethnicity category, ‘Other’ includes participants who identified as Aboriginal or Torres Strait Islander, Native American, Native Hawaiian or Pacific Islander, mixed race, or those who were non-Hispanic and did not specify their race or ethnicity. Hypertension was defined as current use of antihypertensive medication or a blood pressure measurement exceeding 140/90 mmHg at study entry. Dyslipidaemia was defined as taking lipid-lowering medication or having a serum cholesterol level ≥ 212 mg/dL (≥5 mmol/L in Australia) or ≥ 240 mg/dL (≥6.2 mmol/L in the US), or a low-density lipoprotein cholesterol level > 160 mg/dL (>4.1 mmol/L).

The most common condition leading to loss of healthspan was diabetes (48%, *n* = 2803; of whom 2045 had prevalent diabetes at recruitment), followed by cancer (28%, *n* = 1623), CVD (12%, *n* = 727), dementia (7.8%, *n* = 456) and death (5.0%, *n* = 294) (see Supplementary Data Appendix 3 for full details).

Table 2 shows the baseline characteristics of the participants according to incident loss of healthspan. Participants with loss of healthspan over the trial period were older [median age: 74.8; interquartile range (IQR): 71.9,79 vs. 73.7; IQR: 71.5, 77.1] and more likely to be male (41.7% vs. 60.0%). Loss of healthspan was more common among individuals who smoked (5.2% vs. 3.3%) and those with obesity (36.2% vs. 26.5%), hypertension (80.2% vs. 71.5%) and pre-frail or frail (non-frail: 62.6% vs. 50.3%). Previous aspirin use was higher among participants with loss of healthspan compared to those without (13.8% vs. 9.7%). Other characteristics such as education, alcohol consumption and dyslipidemia were similar for participants with and without loss of healthspan.

**Figure 2:**
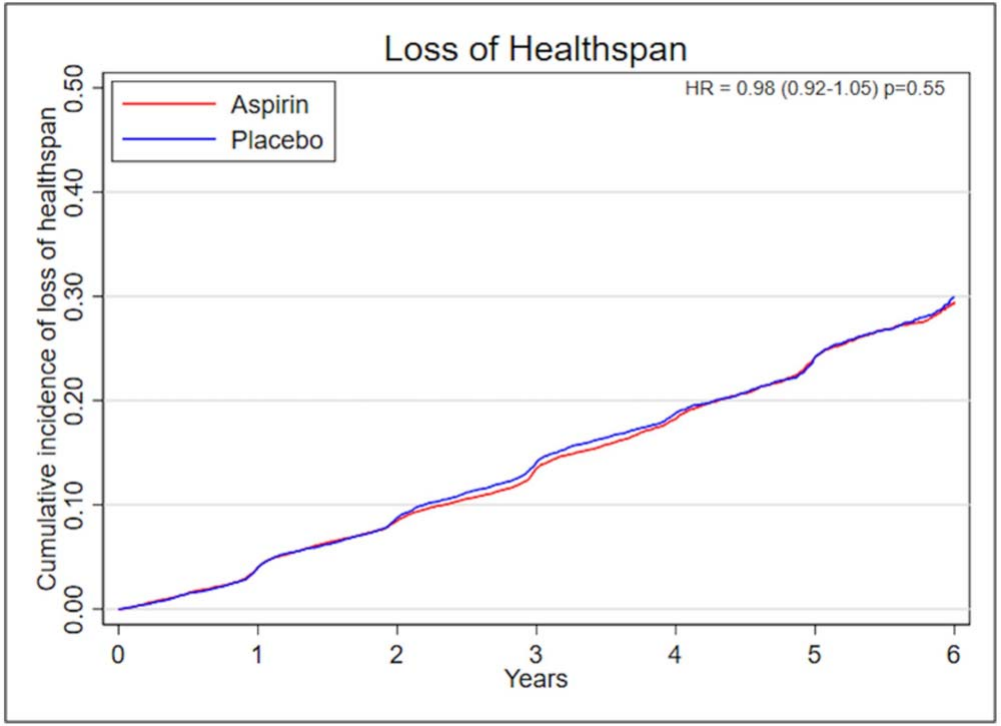
Cumulative incidence of loss of healthspan.

**Figure 3:**
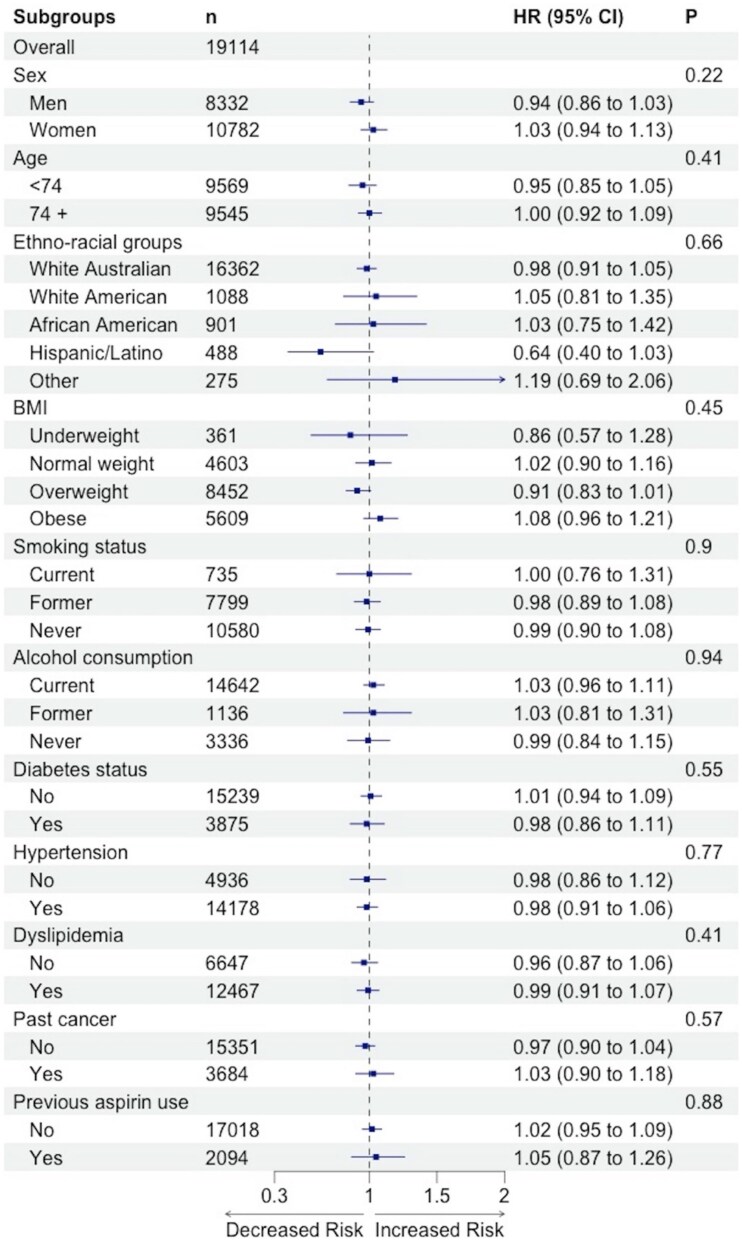
Forest plot of aspirin versus placebo effects on loss of healthspan in subgroups. HR: Hazard ratio; 95% CI: 95% confidence interval; P: P value for interaction term.

By the end of the trial period, the number of years that participants were at risk of loss of healthspan was 35 333 person-years for the aspirin group and 35 480 person-years for the placebo group. There was no significant difference in the number of events (2896 in the aspirin group with a rate of 82.1 events per 1000 person-years and 2939 in the placebo group with a rate of 82.7 events per 1000 person-years) or risk of loss of healthspan between the aspirin group and the placebo group [hazard ratio (HR): 0.98, 95% confidence interval (95% CI): 0.92–1.05] (Figure 2). There was no evidence for significant interactions between subgroups and treatment assignment during the evaluation of potential variations across subgroups (Figure 3). In stratified analyses, the effect of aspirin versus placebo was consistent across all subgroups, though the small group of Hispanic/Latino participants (*n* = 488, HR: 0.64, 95% CI: 0.40–1.03) and overweight individuals (HR: 0.91, 95% CI: 0.83–1.01) appeared to benefit more than others (Figure 3).

The findings from all sensitivity analyses were highly consistent with the main outcome. No significant difference in healthspan was observed between the aspirin and placebo groups from the sensitivity analysis excluding the 2045 participants with diabetes at baseline (HR: 0.98, 95% CI: 0.92–1.05, *P* = .56), the analysis additionally including persistent physical disability as a component (HR: 1.00, 95% CI: 0.94–1.07, *P* = 0.99), or the analysis without diabetes as a component (HR: 0.97, 95% CI: 0.92–1.04, *P* = .41) (see Supplementary Data Appendix 4  for  full  details).

## Discussion

This is the first trial to evaluate the effect of aspirin in older adults on healthspan, defined as time without CVD, cancer, diabetes, dementia, or death. No significant difference in the risk of loss of healthspan was observed between the low-dose aspirin and placebo groups among the participants enrolled in the ASPREE trial. This finding remained unchanged after excluding individuals with prevalent diabetes at baseline and using slightly modified definitions of loss of healthspan (including persistent physical disability, and another without diabetes as a component). Together, these results indicate that daily low-dose aspirin use in late life is not effective at preserving or extending healthspan.

Aspirin has been proposed to reduce disease risk and promote healthy ageing by attenuating systemic inflammation [10, 11, 13, 17, 18]. However, ageing is driven by multiple interconnected pathways beyond inflammation [52], such as metabolism and regulation [e.g. AMPK (a serine/threonine protein kinase) pathway], cellular maintenance and repair mechanisms (e.g. the sirtuin signalling pathway) and growth and nutrient-sensing pathways [e.g. the mTOR (mammalian target of rapamycin) signalling pathway] [52, 53]. Focusing solely on inflammatory pathways may be insufficient to prolong healthspan. Also, the dosage of 100 mg daily aspirin used in this trial may be insufficient to affect chronic disease pathways. While low-dose aspirin has cardioprotective and antiplatelet effects through inhibition of platelet COX-1, higher doses may be required to reduce inflammation [54]. Evidence on the effect of low-dose aspirin on inflammatory biomarkers remains inconclusive, with some studies reporting reductions and others finding no clear association [55, 56]. Previous research also suggested dose-dependent effects, with higher-dose aspirin (300 mg) associated with lower levels of inflammatory markers such as C-reactive protein and interleukin-6 compared to lower doses (150 mg) [57]. Low-dose aspirin could also potentially prolong healthspan through a reduction of cardiovascular events. However, any potential benefits of aspirin must be considered alongside the increased risk of major bleeding reported previously in the ASPREE trial [29], which may outweigh potential gains in otherwise healthy older populations. Furthermore, most participants in the study had not used aspirin prior to the trial and thus were initiating it relatively late in life (age ≥ 70). At that stage, there may be age-related physiological changes and accumulated cellular damage that is more difficult to modify with aspirin treatment.

Our conclusion that aspirin did not extend healthspan aligns with the main findings from the ASPREE trial, with the primary outcome being disability-free survival [58, 59]. No significant short- or long-term (>10 years follow-up) effect of aspirin was observed on the composite measure of life years lived without dementia or persistent physical disability [58, 59]. Our study extends this evidence by incorporating a broader range of incident non-fatal age-related chronic conditions whose absence is central to maintaining healthspan [3, 5, 42, 60].

Subgroup analyses showed no significant heterogeneity across the 11 factors examined, although individuals who were overweight or Hispanic/Latino appeared to derive a potential benefit from aspirin. These findings should be interpreted with caution given the increased risk of Type I error associated with multiple comparisons [61]. This pattern is consistent with previous results from Phyo *et al*. [62], who also observed lower HRs with aspirin for the Hispanic/Latino subgroup for reduced risk of gait slowness and better maintenance of handgrip strength. These findings indicate that the effect of aspirin on healthspan may differ by ethno-racial groups. Hispanics/Latinos had a higher prevalence of metabolic syndromes, including hypertension, dyslipidemia and hyperglycemia [63]. These conditions are potential risk factors for chronic diseases such as CVD and diabetes, which would lead to shorter healthspan [64]. Importantly, among the small proportion of participants (11%) who regularly used aspirin before enrolment, no reduction in the risk of healthspan loss was observed compared with those who initiated aspirin at randomisation. This suggests that prolonged or earlier initiation of low-dose aspirin does not provide additional protection against major chronic diseases, or overall loss of healthspan.

Diabetes was the most common condition leading to loss of healthspan in this study, though cancer was the most prevalent among incident conditions during the trial. These findings are broadly consistent with national data, which ranks diabetes as the fifth most common chronic condition [65]. CVD was less common than cancer, likely reflecting the exclusion of individuals with baseline CVD [66], as the ASPREE trial was intended to evaluate aspirin for the primary prevention of CVD [43]. Cancer incidence increases substantially with age and peaks around the age of 70 [67]; this explains its high prevalence in this cohort. Death was the least frequent contributor to loss of healthspan. This observation is consistent with the recognised healthspan-lifespan gap [3, 68], where people would be likely to live with chronic conditions for a few years before death [3, 68].

We reported that males were more likely to experience loss of healthspan, which is consistent with previous research [68]. Garmany et al. reported a notable healthspan-lifespan gap between sexes [68]. Females generally live longer but experience a higher prevalence of non-fatal chronic conditions and poor health in later life [69–72]. Lifestyle and behavioural factors, particularly smoking and alcohol use, also contribute to sex-specific differences in morbidity and mortality [69–71]. However, unlike Garmany *et al*. who reported that females live longer with disease [68], we observed fewer women experiencing loss of healthspan. A plausible explanation for this variation is the different definitions of healthspan. Garmany *et al*. used health-adjusted life expectancy without disability, whereas our measure was based on predominantly fatal conditions (e.g. cancer, CVD), which are more common in men at the population level [69, 70].

As well-established contributors to poor health [73, 74], smoking and obesity were associated with loss of healthspan in our study. The higher proportion of participants who were smokers or obese among those experiencing loss of healthspan aligns with the findings from the UK Biobank cohort [75]. The authors found healthier lifestyle behaviours (non-smoking, lower alcohol intake, moderate physical activity, healthy BMI, and balanced diet) were linked to lower risk of healthspan termination [75]. Smoking and BMI outside the normal range (18.5–24.9) were independently associated with shorter healthspan [75]. Obesity also increases the risk of hypertension and cancer [76, 77], which reflects our observation that participants with obesity and hypertension were more likely to experience loss of healthspan. Hypertension is a major risk factor for CVD and links to other age-related conditions such as dementia [78, 79]. Overall, these findings reinforce that obesity contributes to loss of healthspan through multiple disease pathways and highlight the potential of lifestyle interventions in extending healthspan and reducing chronic disease burden.

This study has several strengths. First, incident cases of the five major conditions, including CVD, cancer, and dementia, were rigorously adjudicated. This minimised information bias from misclassification, and enhanced outcome accuracy [80]. The randomised, placebo-controlled design of ASPREE and its large cohort of community-dwelling older adults reduced confounding and enabled robust evaluation of low-dose aspirin on healthspan [81]. Additionally, we examined the effect of aspirin use on a composite measure of healthspan loss that incorporated both major chronic conditions and functional limitations. This extends beyond commonly used definitions of healthspan that focus solely on disease outcomes and provides a broader assessment of healthy ageing and overall functional wellbeing. To our knowledge, this is the first study to examine participant characteristics associated with loss of healthspan, offering new insights into risk profiles among older adults.

Our study has several limitations. Loss of healthspan was not a main outcome of the ASPREE trial and we did not include COPD, which was part of previously used definitions [42]. However, post-trial [82], only 43 new cases of COPD or emphysema were reported, highlighting the low incidence in this population of individuals surviving to 70 and being eligible for this trial [43, 83, 84]. There is currently no standardised definition for healthspan [45], but we used the most commonly employed definition covering five major chronic diseases. Functional decline (persistent physical disability) was also explored in subsequent sensitivity analyses. ASPREE participants were relatively healthy at baseline, being free of major chronic disease and disability [85]. However, they did have a high prevalence of chronic conditions such as obesity (30%), hypertension (74%), and dyslipidaemia (65%), which reflects their advanced age and is consistent with prior data [86–90].

## Conclusion

Daily low-dose aspirin did not prolong healthspan in community-dwelling older individuals. This supports existing guidelines that caution against aspirin use in older adults for primary prevention [41]. Further research should consider alternative strategies for promoting healthspan, including integrated approaches with lifestyle, behavioural and pharmacological modifications [91].

## Supplementary Material

aa-26-0973-File002_afag218

## Data Availability

Data from the ASPREE study are available to qualified researchers upon reasonable request, subject to approval by the Principal Investigators and completion of a standard data-sharing agreement. Further details on accessing the data are available via the ASPREE Access Management Site (AMS) (https://ams.aspree.org).
