# Supplementary material for: Effect of aspirin on healthspan in community-dwelling older adults: the ASPirin in reducing events in the elderly study
Source: Age Ageing. 2026 Jul 24;55(7):afag218. doi: 10.1093/ageing/afag218 (PMC13398386; doi:10.1093/ageing/afag218)
Supplement: aa-26-0973-File002_afag218 [file aa-26-0973-file002_afag218.docx]

**Supplementary Data**

**Effect of aspirin on healthspan in community-dwelling older adults: the ASPirin in Reducing Events in the Elderly Study**

**Contents List**

| Appendix 1 | Adjudication of major chronic conditions in loss of healthspan |
| --- | --- |
| Appendix 2 | Number of participants with loss of healthspan at each annual visit in aspirin and placebo groups. |
| Appendix 3 | Number of participants reaching each component of the healthspan outcomes in aspirin and placebo groups. |
| Appendix 4 | Cumulative incidence of modified loss of healthspan: a. loss of healthspan excluding participants with baseline diabetes; b. loss of healthspan including disability as a component, c. loss of healthspan without diabetes as a component. |

**Appendix 1:** **Adjudication of major chronic conditions in loss of healthspan**

Diabetes mellitus status was determined at each annual visit based on a participant’s fasting blood glucose (FBG) level of 7 mmol/L or higher (≥126mg per decilitre) (criteria from the American Diabetes Association (ADA) [1] and World Health Organisation (WHO) [2]), or use of glucose-lowering medication, or self-reported diabetes. Medication use was assessed at baseline and annual visits by verification of prescription medications or written prescriptions brought to the visit. If these were not available, medication use was self-reported and, where possible, verified through review of primary care practice records. The date of diagnosis of diabetes was the date of the annual visit when the participant first self-reported their diagnosis of diabetes, started to use glucose-lowering medication, or a FBG of ≥ 7 mmol/L. Participants with self-reported diabetes at recruitment were also eligible for the trial according to the inclusion criteria [3].

Cardiovascular disease (CVD) was defined as cardiovascular events including a) coronary heart disease death, b) non-fatal myocardial infarction (MI), c) fatal and non-fatal stroke, d) hospitalization for heart failure. Adjudication was based on medical records including clinical and hospitalization records, physicians, and death certificates. The time of CVD diagnosis was defined as the date of the first qualifying cardiovascular event. For non-fatal events, this was the date of the pathology report confirming myocardial infarction, the date of first documented evidence of cerebral dysfunction for stroke, or the date of hospitalisation for heart failure. For fatal coronary heart disease or other cardiovascular deaths occurring without a preceding non-fatal diagnosis, the date of death was used.

The diagnosis of dementia was adjudicated according to the criteria of the Diagnostic and Statistical Manual of Mental Disorders, fourth edition [4]. Specifically, suspected dementia (triggers) was defined as a 3MS (Modified Mini-Mental State examination) score lower than 78, a decrease in 3MS score more than 10.15 points compared to the 6-yearpredicted score adjusted for age and education [5], a clinician/specialist report of possible dementia, or prescription of cholinesterase inhibitors. Six weeks post trigger, all suspected dementia cases had additional assessments, including the Alzheimer’s Disease Assessment Scale–Cognitive subscale [6], Color Trails [7], Lurian overlapping figures [8], and the Alzheimer Disease Cooperative Study Activities of Daily Living scale [9]. Other evidence was also used by the dementia adjudication committee, comprised of international experts, who reviewed all information together with brain CT or MRI, laboratory tests and blood tests to diagnose dementia according to DSM-IV criteria. The date of diagnosis of dementia was recorded as the date of the first valid evidence of suspected dementia or prescription for medication.

Cancer was initially detected through participant’s self-report or review of their medical records. All diagnoses were confirmed by an adjudication committee using histopathological evidence. In instances where histology was not performed, adjudication was based on imaging or clinical evidence, including hospitalization and treatment records [10]. A prior history of cancer was not an exclusion criterion, as the trial excluded only individuals with major illnesses expected to be fatal within five years. All participants, including those with a previous cancer diagnosis, were in good health at enrolment and were randomly assigned to the treatment groups. A history of cancer was not included in the definition of incident loss of healthspan.

Death was mostly identified during the normal trial visits and telephone contact [3]. In the case of failure to contact the participant, health records of the participant or the Ryerson Index in Australia or the National Death Index in the US were reviewed. Two independent sources were used to confirm the notification of death, including from the family, the healthcare physician, or a public death notice. The time of death was recorded as the exact date of death according to the source of evidence.

**Appendix 2:** Number of participants with loss of healthspan at each annual visit in aspirin and placebo groups.

|  | Loss of healthspan | | |
| --- | --- | --- | --- |
|  | Overall | Aspirin | Placebo |
| Baseline | 2045 | 1024 | 1021 |
| Year 1 | 2750 | 1376 | 1374 |
| Year 2 | 3532 | 1755 | 1777 |
| Year 3 | 4336 | 2134 | 2202 |
| Year 4 | 4988 | 2470 | 2521 |
| Year 5 | 5483 | 2721 | 2762 |
| Year 6 | 5773 | 2868 | 2905 |
| Year 7 | 5835 | 2896 | 2939 |

**Appendix 3**: Number of participants reaching each component of the healthspan outcomes in aspirin and placebo groups.

| **Components of loss of healthspan** | **Aspirin** | **Placebo** |
| --- | --- | --- |
| Loss of healthspan* | 2896 | 2939 |
| Diabetes | 1377 | 1426 |
| CVD** | 357 | 370 |
| Coronary heart disease death | 29 | 29 |
| Non-fatal myocardial infarction | 135 | 145 |
| Fatal and non-fatal stroke | 147 | 146 |
| Hospitalization for heart failure | 55 | 57 |
| Dementia | 228 | 228 |
| Cancer | 829 | 803 |
| Death | 143 | 151 |

*There were 78 participants with more than one event of the loss of healthspan, in which 39 in aspirin, 39 in placebo group and one had CVD, cancer and death detected at the same time. Thus, the sum of the number of events for the components of loss of healthspan does not equal the total loss of healthspan events.

**There was one participant with myocardial infarction, stoke and coronary death recorded at the same time in the placebo group, and 14 with myocardial infarction and coronary death at the same time in the aspirin (n=9) and placebo group (n=5). Thus, the total number of CVD events is less than the sum of the individual components.


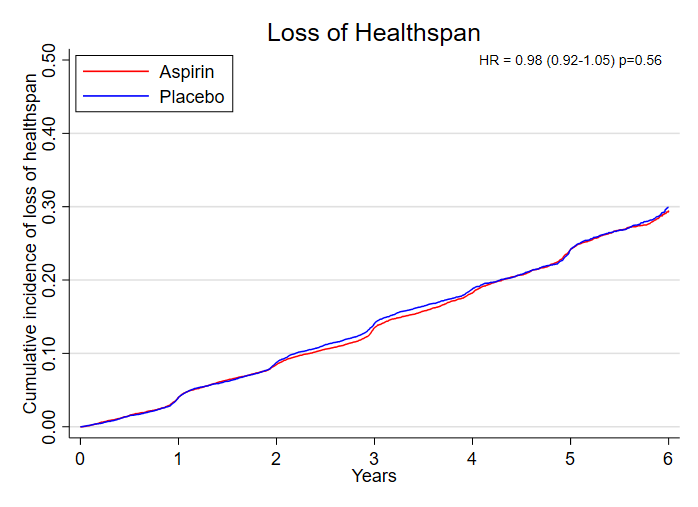


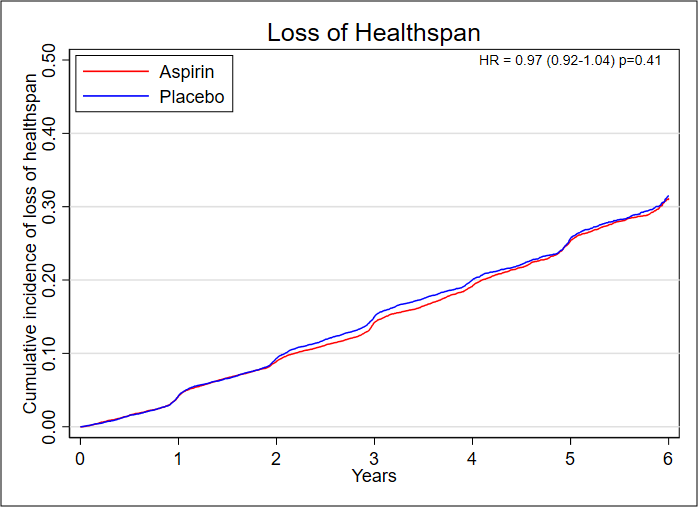


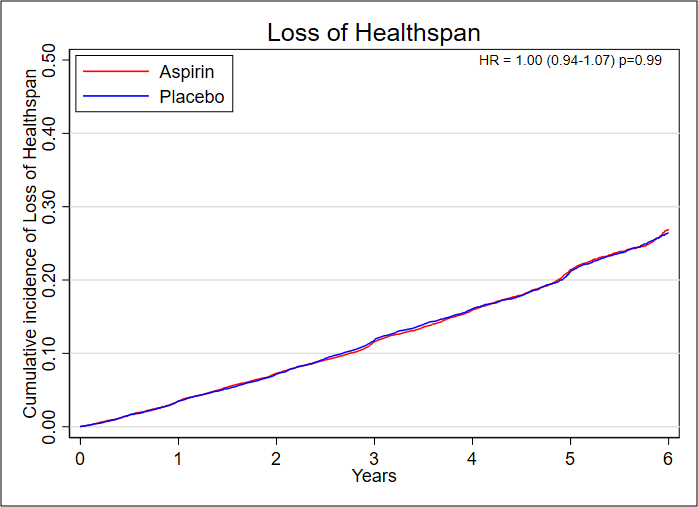


**Appendix 4:** Cumulative incidence of modified loss of healthspan: a. loss of healthspan excluding participants with baseline diabetes; b. loss of healthspan including disability as a component, c. loss of healthspan without diabetes as a component.

**References**

1. Association AD. Classification and Diagnosis of Diabetes: Standards of Medical Care in Diabetes—2021. 2021 [cited 2025]; Available from: <https://diabetesjournals.org/care/article/44/Supplement_1/S15/30859/2-Classification-and-Diagnosis-of-Diabetes>.

2. WHO. Classification of diabetes mellitus. 2019; Available from: <https://www.who.int/publications/i/item/classification-of-diabetes-mellitus>.

3. Group AI. Study design of ASPirin in Reducing Events in the Elderly (ASPREE): a randomized, controlled trial. Contemp Clin Trials. 2013 Nov;36(2):555-64.

4. Diagnostic and statistical manual of mental disorders, 4th ed. Arlington, VA, US: American Psychiatric Publishing, Inc.; 1994.

5. Tombaugh TN. Test-retest reliable coefficients and 5-year change scores for the MMSE and 3MS. Arch Clin Neuropsychol. 2005 Jun;20(4):485-503.

6. Graham DP, Cully JA, Snow AL, Massman P, Doody R. The Alzheimer's Disease Assessment Scale-Cognitive subscale: normative data for older adult controls. Alzheimer Dis Assoc Disord. 2004 Oct-Dec;18(4):236-40.

7. Tyburski E, Karabanowicz E, Mak M, Lebiecka Z, Samochowiec A, Pełka-Wysiecka J, et al. Color Trails Test: A New Set of Data on Cognitive Flexibility and Processing Speed in Schizophrenia. Front Psychiatry. 2020;11:521.

8. Alegret M, Boada-Rovira M, Vinyes-Junqué G, Valero S, Espinosa A, Hernández I, et al. Detection of visuoperceptual deficits in preclinical and mild Alzheimer's disease. J Clin Exp Neuropsychol. 2009 Oct;31(7):860-7.

9. Fish J. Alzheimer’s Disease Cooperative Study ADL Scale. In: Kreutzer JS, DeLuca J, Caplan B, editors. Encyclopedia of Clinical Neuropsychology. New York, NY: Springer New York; 2011. p. 111-2.

10. McNeil JJ, Woods RL, Nelson MR, Reid CM, Kirpach B, Wolfe R, et al. Effect of Aspirin on Disability-free Survival in the Healthy Elderly. N Engl J Med. 2018 Oct 18;379(16):1499-508.
